# Genome-wide DNA methylation measurements in prostate tissues uncovers novel prostate cancer diagnostic biomarkers and transcription factor binding patterns

**DOI:** 10.1186/s12885-017-3252-2

**Published:** 2017-04-17

**Authors:** Marie K. Kirby, Ryne C. Ramaker, Brian S. Roberts, Brittany N. Lasseigne, David S. Gunther, Todd C. Burwell, Nicholas S. Davis, Zulfiqar G. Gulzar, Devin M. Absher, Sara J. Cooper, James D. Brooks, Richard M. Myers

**Affiliations:** 1 HudsonAlpha Institute for Biotechnology, 601 Genome Way, Huntsville, AL 35806 USA; 2 Department of Genetics, Kaul Human Genetics Building, Suite 230, 720 20th Street South, Birmingham, AL 35294 USA; 3 Department of Urology, Stanford University Medical Center, Room S287, 300 Pasteur Drive, Stanford, CA 94305-5118 USA; 4 Present Address: TRM Oncology, 5901-C Peachtree Dunwoody Rd, Suite 200, Atlanta, GA 30328 USA; 5 Present Address: University of Southern California, University Park, Los Angeles, CA 90089 USA; 6 Present Address: Boeing Co., 499 Boeing Blvd, SW, Huntsville, AL 35824 USA; 7 Present Address: Duke University, 101 Science Drive, Durham, NC 27708 USA; 8 Present Address: NuGEN technologies, 201 Industrial Rd #310, San Carlos, CA 94070 USA

**Keywords:** DNA methylation, Prostate cancer, EZH2, Biomarker, Diagnostic

## Abstract

**Background:**

Current diagnostic tools for prostate cancer lack specificity and sensitivity for detecting very early lesions. DNA methylation is a stable genomic modification that is detectable in peripheral patient fluids such as urine and blood plasma that could serve as a non-invasive diagnostic biomarker for prostate cancer.

**Methods:**

We measured genome-wide DNA methylation patterns in 73 clinically annotated fresh-frozen prostate cancers and 63 benign-adjacent prostate tissues using the Illumina Infinium HumanMethylation450 BeadChip array. We overlaid the most significantly differentially methylated sites in the genome with transcription factor binding sites measured by the Encyclopedia of DNA Elements consortium. We used logistic regression and receiver operating characteristic curves to assess the performance of candidate diagnostic models.

**Results:**

We identified methylation patterns that have a high predictive power for distinguishing malignant prostate tissue from benign-adjacent prostate tissue, and these methylation signatures were validated using data from The Cancer Genome Atlas Project. Furthermore, by overlaying ENCODE transcription factor binding data, we observed an enrichment of enhancer of zeste homolog 2 binding in gene regulatory regions with higher DNA methylation in malignant prostate tissues.

**Conclusions:**

DNA methylation patterns are greatly altered in prostate cancer tissue in comparison to benign-adjacent tissue. We have discovered patterns of DNA methylation marks that can distinguish prostate cancers with high specificity and sensitivity in multiple patient tissue cohorts, and we have identified transcription factors binding in these differentially methylated regions that may play important roles in prostate cancer development.

**Electronic supplementary material:**

The online version of this article (doi:10.1186/s12885-017-3252-2) contains supplementary material, which is available to authorized users.

## Background

Currently, the most frequently used methods for detecting prostate cancer are a digital rectal exam and a blood test to determine levels of prostate-specific antigen (PSA) produced by the prostate gland [1]. However, these diagnostic tools can lack the sensitivity required to detect very early prostate lesions [2]. Furthermore, PSA levels can increase for reasons unrelated to cancer or not increase when cancer is present [2]. If a prostate cancer is suspected, prostate biopsies are performed. However, prostate biopsies are invasive, and can lead to false-negatives and repeat biopsies, as they do not sample the entire prostate. Recent developments in prostate cancer detection include measuring the non-coding RNA prostate cancer antigen 3 (*PCA3*) and transmembrane protease, serine 2 (*TMPRSS2*):v-ets erythroblastosis virus E26 oncogene homolog (avian) (*ERG*) gene fusion in urine to identify patients requiring repeat biopsies despite an initial negative biopsy [3–5]. However, there is a clear need to identify novel biomarkers for diagnostic purposes that are sensitive and specific to prostate cancer.

Epigenetic patterns are known to be altered in several different cancer types, including prostate cancer, and signatures of DNA methylation may serve as potential diagnostic or prognostic biomarkers [6]. Cancer-derived, methylated DNA has been identified and purified from both patient serum and urine, making it a promising option for a non-invasive biomarker [7]. Previous studies investigating DNA methylation patterns at select genomic loci in prostate cancer resulted in discoveries of epigenetic differences between prostate cancer tissue and benign-adjacent prostate in genes such as glutathione s-transferase 1 (*GSTP1*), Ras association domain family member 1 (*RASSF1*), and adenomatous polyposis coli (*APC*), among others [8–10]. Recently, there have been studies using global approaches in prostate cancer that have identified DNA methylation alterations in malignant prostate tissue, including a previous study from our group [11–17]. We sought to expand upon our previous discoveries by performing genome-wide measurements of DNA methylation in 73 clinically annotated fresh-frozen prostate cancers and 63 benign-adjacent prostate tissues using the Illumina Infinium HumanMethylation450 BeadChip array, which offers greater genomic coverage compared to the Methyl27 array that we previously used [11]. We present here novel DNA methylation-based diagnostic models, and discuss transcription factors whose binding sites are enriched in regions of differential methylation in prostate cancer.

## Methods

### Tissue collection and nucleic acid extraction

We collected the prostate cancer and benign-adjacent tissues used in this study at Stanford University Medical Center between 1999 and 2007 from patients undergoing radical prostatectomy with patient informed consent under an IRB-approved protocol. The percentage of prostate cancer epithelial cells in each sample was assessed by a pathologist specializing in genitourinary cancers on hematoxylin and eosin (H & E) stained frozen sections of the tissues from which the DNA was extracted. We selected those samples in which at least 90% of the epithelial cells were cancerous for nucleic acid extractions, and used the QIAGEN AllPrep DNA/RNA mini kit (QIAGEN) to extract DNA and RNA.

### DNA methylation analysis via Illumina Infinium HumanMethylation 450 K

We assayed DNA methylation levels by using the Illumina Infinium HumanMethylation 450 K beadchip array (Illumina, San Diego, CA, USA) [18] and calculated the methylation beta score as: b = Intensity_Methylated_/(Intensity_Methylated_ + Intensity_Unmethylated_). We converted data points that were not significant above background intensity to NAs. We removed CpGs having greater than 10% missing values prior to normalization. Data was normalized with the ComBat R package [19]. Post-ComBat normalization, we observed that the Infinium I and II assays showed two distinct bimodal b-value distributions, so we developed a regression method to convert the type I and type II assays to a single bimodal b-distribution corresponding to Reduced Representation Bisulfite Sequencing (RRBS) b-values [20]. After the Methylation 450 K data was converted to RRBS b-values, any values less than zero were assigned zeros and values greater than one were assigned ones. The equations for correction are shown below:

Infinium I to RRBS:

$$ {\mathrm{RRBS}}_{\upbeta}=0.00209+0.4377 \times {\mathrm{Methyl}450}_{\upbeta}+0.6303\times {{\ \mathrm{Methyl}450}^2}_{\upbeta} $$

Infinium II to RRBS:

$$ {\mathrm{RRBS}}_{\upbeta}=\hbox{-} 0.01146+0.2541 \times {\mathrm{Methyl}450}_{\upbeta}+0.9832\times {{\ \mathrm{Methyl}450}^2}_{\upbeta} $$

### Linear mixed model and logistic regression analysis

Linear mixed model analysis of the methylation data was performed using the lme command in R, with patient as a random effect, and age and ethnicity as fixed effects. Logistic regression was performed using the glm command (family = binomial). The *p*-values were adjusted using the Benjamini and Hochberg method [21]. CpGs with a standard deviation of less than 1% across samples were removed prior to analysis.

### RNA-seq library construction and differential expression analysis

We constructed RNA sequencing libraries using a transposase-mediated construction method described previously [22]. Four RNA-seq libraries were pooled into each lane and sequenced using Illumina HiSeq 2000 instruments to generate paired-end 50 sequencing reads (Illumina, San Diego, CA, USA). Read-pairs were aligned to Gencode (version 9.0) using TopHat (version 1.4.1), and the relative abundance of each transcript was quantified using Cufflinks (version 1.3.0) and BEDTools [23–26]. Differential expression analysis was conducted based on tumor status using DESeq2 (version 1.8.1) with default settings in likelihood ratio test (LRT) mode. Transcripts from the X and Y-chromosomes were removed prior to differential expression analysis.

### Pathway enrichment analysis

Chromosomal positions of significant CpGs were annotated using RefSeq (hg19 assembly) [27]. The Gene Set Enrichment Analysis (GSEA) tool was used to analyze enriched cellular pathways [28]. GSEA was run with Kegg and Reactome selected, and used an FDR-corrected q-value cutoff of 0.05.

### Hierarchical clustering

Hierarchical clustering was performed using Cluster 3.0 [29]. Data was mean-centered and clustered by both gene and array using Euclidean distance with average linkage. Clusters were visualized using TreeView [30].

### TCGA data

TCGA DNA methylation (Illumina Methylation 450 k) datasets and associated clinical data for prostate (PRAD_2013_09_07), lung (LUAD_2013_09_07), breast (BRCA_2013_09_07) and pancreatic (PAAD_2013_09_07) tissues were downloaded from the UCSC cancer genome browser at time of manuscript preparation. Datasets were normalized prior to validation analysis.

### Transcription factor overlap

ENCODE transcription factor binding data was downloaded from http://genome.ucsc.edu/cgi-bin/hgTrackUi?db=hg19&g=wgEncodeRegTfbsClusteredV3. We overlapped the CpGs found within gene regulatory regions (promoter, first exon or first intron) from the top 10,000 most significant CpGs from regression analysis with the ENCODE transcription factor binding sites, and used a Fisher’s exact test to determine transcription factor binding sites enriched for differential methylation over background. For EZH2 binding site overlap, we overlapped significant CpGs (FDR *p*-value < 0.05) with EZH2 binding data previously published [31]. For gene expression analysis, genes that were differentially expressed between tumor and normal (DESeq2-based FDR *p*-value < 0.05) were designated as overlapping a TF binding site if greater than 50% the binding site fell within the transcript promoter region. The promoter region was defined as 1000 bp upstream to 500 bp downstream of the transcription start site. Transcription factors with a Bonferroni-corrected *p*-value <0.05 were classified as significantly enriched.

## Results

### Identification of differentially methylated cytosines in prostate cancer

**Fig. 1:**
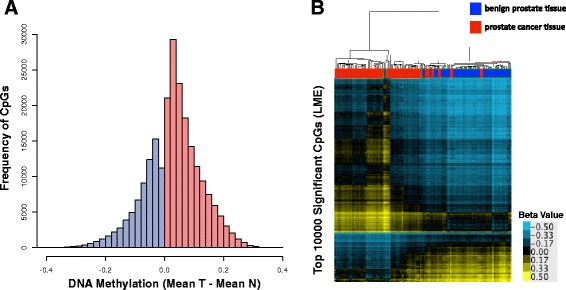
**a** Histogram of differentially methylated CpGs (LME, FDR < 0.05). Blue represents CpGs that have significantly higher methylation in benign-adjacent prostate tissue when compared to prostate cancer tissues (73,912 CpGs), and red represents CpGs that have significantly higher methylation in prostate cancer tissues (152,324 CpGs). **b** Heatmap of the top 10,000 CpGs with the most statistically significant DNA methylation differences between unaffected prostate tissue and prostate cancer tissue based on LME *p*-value. Color bar represents beta score with 0.5 subtracted

To investigate differential DNA methylation associated with prostate cancer, we used the Illumina Infinium HumanMethylation450 BeadChip Methylation Assay, which covers more than 485,000 CpGs located throughout the human genome [18]. DNA methylation patterns were measured in 73 patient prostate cancer tissues and 63 benign-adjacent tissues, 52 of which are patient-matched (Table 1). Mixed model linear regression analysis identified (LME) 226,235 CpGs with significantly different methylation levels (LME, FDR-adjusted *p*-value <0.05) in cancer tissues compared to benign-adjacent prostate tissues. Of the 226,235 significant CpGs, ~67% had increased methylation and ~33% had decreased methylation in the cancer tissues compared to the benign-adjacent tissues (Fig. 1a). CpGs with higher methylation levels in tumor tissues were more likely to be within CpG islands (Fisher’s Exact Test, *p*-value 3.44e–154, OR = 1.18, 95% CI = 1.18–1.12), and statistically significant CpGs were also found in greater proportion in gene regulatory regions (promoter, first exon, or first intron) than in gene body regions (other exon, other intron, or 3′ proximal region), although this association did not reach statistical significance. (Table 2A, B).

**Table 1:** Clinical data for patients used in this study

	Training cohort	Testing cohort (TCGA)
Patients [n]		
Tumor tissues	73	213
benign-adjacent tissues	63	49
patient-matched tissues	52	49
Age		
Mean [years]	59.9	60.4
Median [years]	61	61
Range age [years]	43–73	43–75
Preoperative PSA [ng/mL]		
Range	0.94–42	1.6–87
Mean	6.8	10.9
Median	5.62	7.4
< 4 [n]	15	19
4–10 [n]	44	100
> 10 [n]	9	55
unknown	5	39
Gleason Grade [n]		
(<7)	16	15
(3 + 4)	40	84
(4 + 3)	10	50
8	4	25
(>8)	2	39
unknown	1	0
T Category		
T2	2	NA
T2a	3	8
T2b	50	2
T2c	NA	82
T3a	8	71
T3b	7	43
T4	1	5
unknown	2	2
Nodal Status		
N0	66	160
N1	5	22
unknown	2	31

**Table 2:** Genomic regions of differentially methylated CpGs

Genomic location	Sig. CpGs more methylated in tumor	Sig. CpGs less methylated in tumor	Fisher’s exact *p*-value
A. Island versus non-island			
CG Island	93,536	35,639	3.44E-154
Non-CG Island	58,787	38,273	
B. Regulatory region versus gene body (All significant CpGs)			
Regulatory Region	55,029	23,056	0.49
Gene Body	46,502	19,858	
C. Regulatory Region Versus Gene Body (Top 10,000 most significant CpGs)			
Regulatory Region	3165	776	1.50E-02
Gene Body	1902	700	

Regulatory Region = promoter, first exon, first intron

Gene Body = other exon, other intron, 3′ proximal

CG Island = CG islands, CG shelves, and CG shores

A) Analysis of CpGs in islands versus non-islands. B) Analysis of CpGs in gene bodies versus gene regulatory regions for all significant CpGs. C) Analysis of CpGs in gene bodies versus gene regulatory regions for the top 10,000 most significant CpGs

To explore the genes and cellular pathways found in differentially methylated regions, we analyzed the top 10,000 most significant CpGs between the prostate cancer tissue and unaffected prostate tissue (LME, FDR *p*-value cutoff of <4.27e-13) (Fig. 1b). Of these, 75% had a higher methylation level in the cancer tissues. We divided the top 10,000 CpGs that were uniquely annotated to one gene by whether they resided in the gene regulatory region (promoter, first exon, and first intron) or the gene body (other exon, other intron, and 3 prime proximal region) and found that the CpGs with higher methylation in the cancer compared to benign tissue were statistically more likely to be associated with a gene regulatory region (Fisher’s Exact Test, *p*-value 0.015, OR = 1.10, 95% CI = 1.018–1.18) (Table 2C).

We used Gene Set Enrichment Analysis (GSEA) to determine which gene pathways are represented in the top 10,000 most significant CpGs [28]. We observed 3165 CpGs in the regulatory regions of 1589 genes with higher DNA methylation in the prostate cancer compared to benign tissue. GSEA analysis of those 1589 genes showed a strong signature for glycosaminoglycan metabolism, with five of the top 10 significantly enriched pathways associated with heparan sulfate metabolism and chondroitin sulfate metabolism (Additional file 1: Table S1). Other pathways included focal adhesion, pathways in cancer, Wnt signaling pathway, developmental biology and axon guidance. The enrichment for glycosaminoglycan metabolism pathways was specific to CpGs in regulatory regions with higher methylation in the cancer. Conversely, there were 776 CpGs located in gene regulatory regions of 621 genes with lower methylation in prostate cancer tissue. GSEA analysis of these genes showed enrichment for olfactory signaling, G-protein coupled receptor signaling, metabolism of carbohydrates, apoptosis, immune system, neuronal growth factor signaling pathway, and hemostasis (Additional file 2: Table S2).

### Overlap of ENCODE transcription factor ChIP-seq data and differential DNA methylation highlights the importance of EZH2 in prostate cancers

**Fig. 2:**
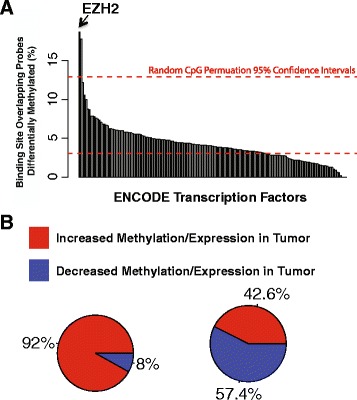
Overlap of top 10,000 most significant (LME *p*-value) DNA methylation sites in gene regulatory regions and higher methylation in prostate cancer tissues with ENCODE transcription factor binding sites highlights the role EZH2 plays in prostate cancer. **a** Barplot showing the relative percent of ENCODE transcription factor binding sites containing significant methylation changes. Dashed red lines represent the upper and lower 95% confidence intervals generated from enrichment values of randomly selected methylation sites. **b** Pie charts demonstrating the directionality of significant DNA methylation sites and gene expression levels within 1 kb of EZH2 binding sites

We compared the DNA methylation data with transcription factor chromatin immunoprecipitation sequencing data (ChIP-seq) measured by the Encyclopedia of DNA Elements (ENCODE) Consortium to test whether there was an enrichment of transcription factor binding sites coinciding with the top 10,000 most differentially methylated CpGs between prostate cancer and benign-adjacent tissues. Enhancer of zeste homolog 2 (EZH2) was the most significantly enriched TF overlapping CpGs with higher methylation in the cancer tissues from our dataset (Fisher’s Exact Test, Bonferroni adj. *p*-value 7.54e-172, OR = 3.4, 95% CI = 3.14–3.68), and this observation was validated in The Cancer Genome Atlas (TCGA) prostate methylation dataset (Fisher’s Exact Test, Bonferroni adj. *p*-value 6.48e-120, OR = 2.48, 95% CI = 2.29–2.69) (Fig. 2a and Additional file 3: Table S3A and B). ENCODE TF binding data was generated from multiple types of cell lines. To determine whether EZH2 binding enrichment occurs in prostate cancer specifically, we compared the significant CpGs differentially methylated between prostate cancer and benign-adjacent tissue (FDR *p*-value cutoff of <0.05) with previously published EZH2 binding events from androgen-dependent (AD) and androgen-independent (AI) cell line models [31]. EZH2 binding events were significantly enriched in both the AD and AI contexts, although we observed a higher level of enrichment in the AI context (Fisher’s Exact Test, AD enrichment *p*-value =0.01, OR = 1.15, 95% CI = 1.01–1.30; AI enrichment *p*-value =0.00013, OR = 1.18, 95% CI = 1.08–1.29) (Additional file 3: Table S3C). Notably, in our tissue cohort, significant CpGs found in proximity to EZH2-bound sites are mostly hypermethylated (Fig. 2b). We also observed that a majority of transcripts that contain EZH2 binding sites in the promoter region that are differentially expressed between prostate cancer tissue and the benign-adjacent tissues have decreased expression in the prostate cancer tissue (Fig. 2b).

For CpGs with lower methylation in the prostate cancer tissues in comparison with the adjacent-unaffected tissue, there were two TFs with significant overlap that were bound in these regions: FOXA2 and SETDB1 (Additional file 3: Table S3D). However, we were unable to validate the enrichment we observed for these TFs in the TCGA prostate methylation dataset (Additional file 3: Table S3E).

### Discovery and validation of most distinguishing DNA methylation sites in prostate tissues

**Fig. 3:**
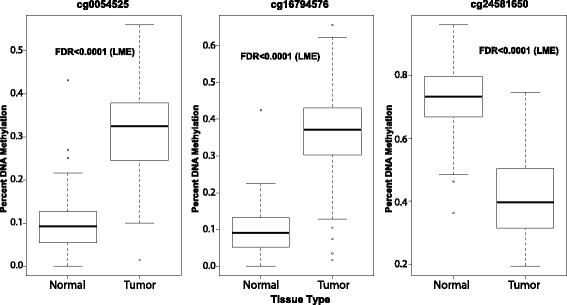
Boxplots of CpGs in the top diagnostic models. Normal data is from benign-adjacent tissues and Tumor Data is from patient cancer tissues

**Fig. 4:**
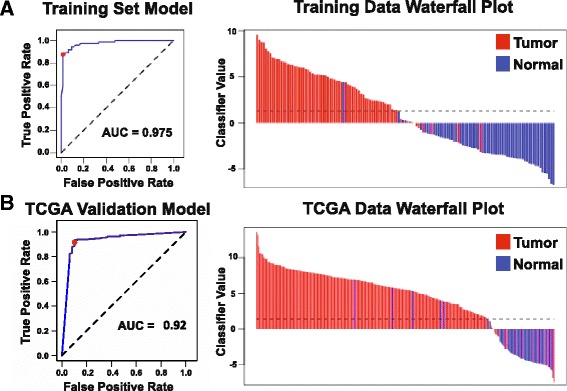
ROC curve and waterfall plots for performance of the top 3 CpG diagnostic model in **a** training and **b** validation datasets. The value of the classifier is given by 6.52–17.04*cg00054525 + 24.18*cg16794576–13.82*cg24581650, where the intercept and coefficients have been regressed by a binomial generalized linear model. A threshold value of this classifier was chosen to yield maximal non-unity specificity in the training set. The red dot on the ROC curve corresponds to the sensitivity and specificity of the classifier at the chosen threshold. The dashed line on the waterfall plots is drawn at the chosen threshold value of the classifier

To discover DNA methylation patterns that best distinguish prostate cancer tissue from benign-adjacent tissue, we performed logistic regression on the 100 most statistically significant CpGs from the linear mixed model regression (FDR *p*-value cutoff of <8.22e-15). We tested each combination of three CpGs within the top 100 most significant CpGs, as models containing three CpGs resulted in the smallest Akaike Information Criterion (AIC) value. We calculated the Area Under the Curve (AUC) for each Receiver Operating Characteristic (ROC) curve to identify the model with a maximal AUC. The top DNA methylation diagnostic model based on AUC from our analysis consists of cg00054525, cg16794576, and cg24581650 (Fig. 3, Additional file 4: Table S4). This DNA methylation model produces a ROC curve with an AUC of 0.97 in our cohort of prostate tissues and has a specificity of 98.4% and a sensitivity of 87.5%, indicating that DNA methylation status at these three genomic positions has very high predictive power for distinguishing malignant tissue from benign tissue (Fig. 4a). The corresponding waterfall plot demonstrates the high accuracy our top DNA model performs in classifying the prostate tissues (Fig. 4a). Based on analysis of methylation data from other tissue types, these methylation differences are unique to prostate cancer cells, as DNA methylation levels at these sites performed poorly in distinguishing lung, pancreatic, and breast cancer tissue from benign tissue (Additional file 5: Figure S1). The top three diagnostic CpGs are in close proximity to four total transcripts based on annotation, including CYBA, ERGIC1, HLA-J, and NCRNA00171. Out of these four transcripts, *ERGIC1* has a statistically significant difference in mRNA expression level between prostate malignant tissue and benign tissue (DESeq2, adj. *p*-value 6.4e-06) (Additional file 6: Figure S2).

We utilized prostate data from TCGA as a validation cohort for our DNA methylation signature (Table 1). The TCGA methylation data was also measured using the Human Methylation 450 BeadChip and included 213 prostate cancer tissues and 49 normal tissues. Our model, based on 3 CpGs, could distinguish normal from malignant prostate tissues with a sensitivity of 84.5% and a specificity of 91.7% in the TCGA dataset, resulting in a ROC curve with an AUC of 0.920 (Fig. 4b, Additional file 4: Table S4). To determine how our top diagnostic model performs in the context of benign prostate hyperplasia (BPH), we used a previously published cohort (GEO accession: GSE55599) to see if our top DNA methylation model could distinguish prostate cancer tissue from prostate tissue obtained from patients with benign-hyperplasia and found that our model could perfectly discriminate these two types of tissues (Additional file 7: Figure S3) [32].

Additionally, we investigated prostate diagnostic markers from significant CpGs from the linear mixed model analysis that exclusively demonstrated an increase in methylation in cancers, as biomarkers that are hypermethylated in the cancer tissues are potentially more easily translatable to the clinic. In this context, the top model consists of cg15338327, cg00054525, and cg14781281 (Additional file 8: Figure S4), resulting in a ROC curve with an AUC of 0.97 in our dataset and an AUC of 0.92 in the TCGA prostate validation dataset (Additional file 9: Figure S5A-B). This hypermethylated diagnostic model also performed well at distinguishing benign-hyperplasia prostate tissue from prostate cancer tissue, with an AUC of 0.85 (Additional file 9: Figure S5C-D).

## Discussion

Shifts in epigenetics play a large role in cancer formation and maintenance, and DNA methylation is a stable modification that can be detected non-invasively in fluids such as urine, blood and saliva. For these reasons, DNA methylation is an attractive cancer biomarker candidate. In our study, we identified a large number of CpG loci with statistically significant DNA methylation levels between our cohort of prostate cancer tissues and the adjacent, unaffected prostate tissues. More than half of the significant CpGs were found to be hypermethylated in the prostate tumor tissues. Our previous work strongly suggests that these methylation changes are the result of dysregulation of the DNA methyltransferases DNMT3A2 and DNMT3B [11].

Global DNA methylation changes implicate genes associated with the stroma and tumor microenvironment as being enriched targets for methylation changes. We observed an overwhelming signature of glycosaminoglycan (GAG) metabolism in the regulatory regions of transcripts with higher methylation in malignant tissues. GAGs are long polysaccharides that have both structural and signaling roles within the extracellular matrix and cellular membranes and have a documented role in many cancers [33]. In prostate cancer, altered expression of GAGs has been observed in early stage prostate cancer and correlates with malignant progression. A large body of literature documents numerous ways that altered proteoglycan metabolism can influence prostate cancer development and progression, including altering prostate cancer cell growth, motility, survival, local diffusion of growth factors, and cell signaling [34]. The enrichment of GAG metabolism, and specifically heparan sulfate and chondroitin sulfate metabolism, in regions with lower DNA methylation in benign-adjacent prostate tissues likely points to the structural changes occurring in the extracellular space surrounding the cancer, and we confirmed that the majority of these genes have higher expression in the benign-adjacent tissues (Additional file 10: Table S5A). A recent study investigating transcriptional activity of genes involved in heparan sulfate biosynthesis in prostate tissues found that these genes have lower expression in prostate cancer tissues compared to prostate tissue from individuals with no prostate cancer, and findings from our study suggest that the expression of these genes is down-regulated in prostate cancer, at least in part, due to epigenetic changes [35].

Regions of the genome with reduced DNA methylation in the prostate cancer tissue were enriched for a diverse collection of cellular pathways. Olfactory signaling was represented among the enriched pathways. We observed a large number of odorant receptor genes had less methylation in their gene regulatory region in the prostate cancer tissue in comparison with benign-adjacent prostate tissue, and their gene expression levels were mostly higher in the cancer tissues (Additional file 10: Table S5B). A recent study demonstrated that activation of odorant receptors increases cell invasion into collagen gel [36].

We overlaid ENCODE TF ChIP-seq data with sites of differential methylation and observed that EZH2 was the most highly enriched TF binding in these regions. EZH2 is part of the polycomb repressive complex that is known to regulate chromatin structure during development primarily through repression of expression of a large and diverse set of genes [37]. EZH2 functions to repress gene expression through methylation of histone H3 at lysine 27 (H3K27 methylation), and EZH2 can also recruit DNA methyltransferases to EZH2-target promoters [31, 38, 39]. EZH2 expression increases throughout prostate cancer progression and EZH2 expression levels are associated with methylation level in prostate cancer [11, 40]. Our data suggest that EZH2-directed methylation alterations are critical for the formation and maintenance of prostate cancer, in addition to roles EZH2 plays in castration-resistant prostate cancer.

A practical application of genome wide DNA methylation profiling is the identification of candidate diagnostic biomarkers. We have demonstrated that as few as three CpGs can be used to distinguish benign-adjacent from malignant prostate tissues with high sensitivity (92.6%) and specificity (87.8%). Methylation biomarkers have been identified in prostate cancer previously, including promoter segments of GSTP1, RASSF1, and APC, which are used in commercial tissue-based test to identify patients needing repeat biopsies after an initial negative biopsy [41]. Clinical validation studies of this commercial methylation-based assay obtained a sensitivity level of 68% and a specificity level of 64% [42, 43]. We also investigated candidate diagnostic models developed from CpGs that have higher methylation in prostate cancer tissue. Hypermethylated CpGs appear to retained throughout all stages of prostate cancer, likely due to selection pressures, whereas CpGs that become hypomethylated in prostate cancer are less likely to be preserved [44]. In this context, we tested a 3-CpG model that provided a sensitivity of 90% and a specificity of 82% that again, exceed those reported for DNA methylation markers currently in use. One of the diagnostic the diagnostic CpGs (cg00054525) falls within the regulatory region of the *CYBA* gene. Methylation of *CYBA* has been previously associated with the progression of melanoma [45, 46]. However, other genes associated with our diagnostic CpGs, such as *HLA-J* and a non-coding RNA, have not yet been associated with cancer, to our knowledge, and thus, introduce new biological aspects to explore. Our model’s diagnostic performance is relatively poor in lung, breast and pancreas adenocarcinomas, suggesting it has some specificity to prostate cancer. This is a characteristic that could hold value in future studies pursuing a non-invasive, peripheral fluids-based assay.

It is important to note that currently available prostate cancer patient cohorts, including our own, are limited in numbers of samples, and future studies will elucidate the value of our DNA methylation signatures across larger cohorts of prostate cancer patients. Furthermore, the full utility of these DNA methylation-based diagnostic biomarkers will be realized when they can be measured in peripheral fluids from patients. Thus, an important future direction of our study is to determine whether these DNA methylation signatures can be detected in patient urine or blood. To definitively determine their clinical relevance, it will be important to directly compare these diagnostic biomarkers to clinically established markers, such as PSA. Finally, given the recent identification of molecular subtypes of prostate cancer, it will be important to determine DNA methylation patterns that not only distinguish tumor tissue from benign tissue, but also can inform about the molecular subtype of the tumor [17].

## Conclusions

Our results indicate that DNA methylation can be used to successfully distinguish prostate cancer tissue from benign-adjacent tissue and that our 3-CpG DNA methylation signatures are not common to other cancers. Sites of differential methylation point to a role for odorant receptors and GAG metabolism and integration of ENCODE transcription factor binding data demonstrates EZH2 enrichment at the sites of altered DNA methylation. These data have the potential to impact both diagnosis and treatment of prostate cancer.

## Additional files

Additional file 1: Table S1. Gene Set Enrichment Analysis. Top 10 enriched pathways from 1589 genes with high methylation in cancer tissue. (XLSX 9 kb)

Additional file 2: Table S2. Gene Set Enrichment Analysis. Top 10 enriched pathways from 621 genes with low methylation in cancer. (XLSX 9 kb)

Additional file 3: Table S3. ENCODE and EZH2 transcription factor overlay significantly associated with regions of differential methylation in cancer tissue. A) Transcription factors enriched in the top 10,000 most significant CpGs found in gene regulatory regions with higher methylation levels in the tumor tissues. B) TCGA validation of transcription factors enriched in the top 10,000 most significant CpGs found within gene regulatory regions with higher methylation levels in the tumor tissues. C) Analysis of significant methylation sites overlaid with EZH2 binding data from an androgen-dependent (LNCaP) and an androgen-independent (LNCaP-abl) prostate cancer cell line. D) Transcription factors enriched in the top 10,000 most significant CpGs within gene regulatory regions with higher methylation in the benign-adjacent tissue. E) TCGA validation of transcription factors enriched in the top 10,000 most significant CpGs found within gene regulatory regions with higher methylation in the benign-adjacent tissue. (XLSX 45 kb)

Additional file 4: Table S4. Coefficients, standard errors and performance statistics for the top diagnostic model and top hypermethylated CpG model. (XLSX 46 kb)

Additional file 5: Figure S1. ROC curve and waterfall plots for diagnostic model applied to a) lung b) pancreatic and c) breast cancer TCGA datasets. (PDF 1091 kb)

Additional file 6: Figure S2. Gene expression of genes in close proximity to the top diagnostic model. (PDF 118 kb)

Additional file 7: Figure S3. ROC curve and waterfall plot for diagnostic model applied to an independent cohort of prostate cancer and benign prostate hyperplasia samples. (PDF 775 kb)

Additional file 8: Figure S4. Boxplots of CpGs in the top diagnostic model from hypermethylated CpGs. Normal data is from benign-adjacent tissues and Tumor data is from patient cancer tissues. (PDF 129 kb)

Additional file 9: Figure S5. ROC curve and waterfall plot for the top hypermethylated CpG model in the TCGA validation cohort (a-b) and BPH cohort (c-d). (PDF 842 kb)

Additional file 10: Table S5. DESeq2 results for gene expression differences between prostate cancer tissue and benign-adjacent tissue for select transcripts with methylation differences that drive GSEA enrichments. A) DESeq2 results for gene expression of glycosaminoglycan metabolism genes with higher methylation in prostate cancer tissue. B) DESeq2 results for gene expression of odorant receptor genes with reduced methylation in prostate cancer tissue. (XLSX 51 kb)

## Abbreviations

AD: Androgen-dependent
AI: Androgen-independent
AIC: Akaike information criterion
APC: Adenomatous polyposis coli
AUC: Area under the curve
ChIP-seq: Chromatin immunoprecipitation sequencing data
ENCODE: Encyclopedia of DNA elements
ERG: v-ets erythroblastosis virus E26 oncogene homolog (avian)
EZH2: Enhancer of zeste homolog 2
FDR: False discovery rate
GSEA: Gene set enrichment analysis
GSTP1: Glutathione s-transferase pi 1
PCA3: Prostate cancer antigen 3
PSA: Prostate-specific antigen
RASSF1: Ras association domain family member 1
ROC: Receiver operating characteristic
RRBS: Reduced representation bisulfite sequencing
TCGA: The cancer genome atlas
TF: Transcription factor
TMPRSS2: Transmembrane protease, serine 2
