# Supplementary figures and images for: Genome-wide DNA methylation measurements in prostate tissues uncovers novel prostate cancer diagnostic biomarkers and transcription factor binding patterns
Source: BMC Cancer. 2017 Apr 17;17:273. doi: 10.1186/s12885-017-3252-2 (PMC5392915; doi:10.1186/s12885-017-3252-2)

**A**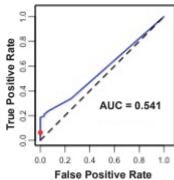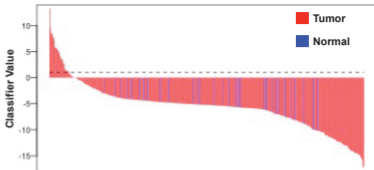**B**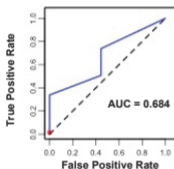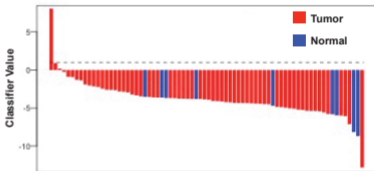**C**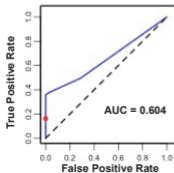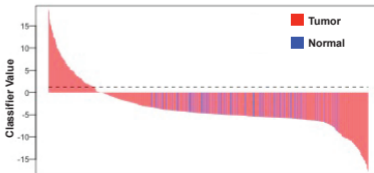

Supplement: Supplementary file 5 — ROC curve and waterfall plots for diagnostic model applied to a) lung b) pancreatic and c) breast cancer TCGA datasets. (PDF 1091 kb) [file 12885_2017_3252_MOESM5_ESM.pdf]

NCRNA00171

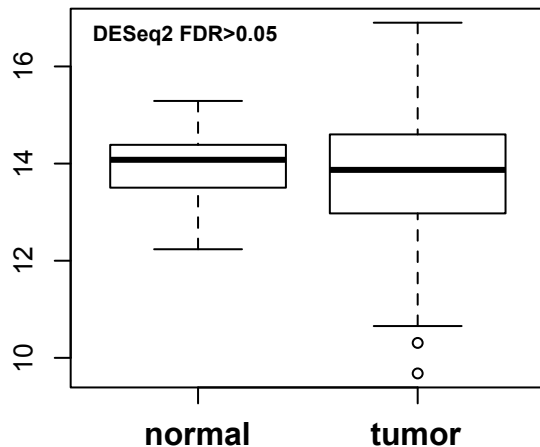

CYBA

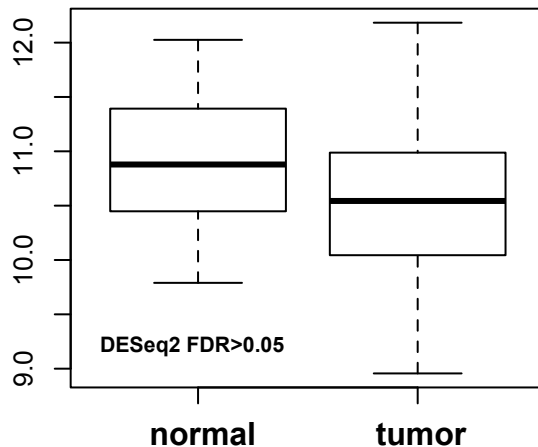

HLA-J

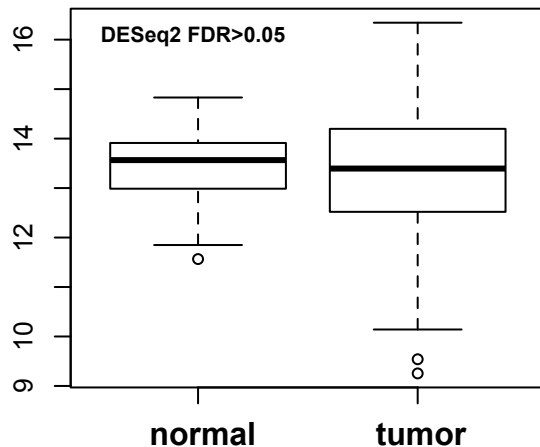

ERGIC1

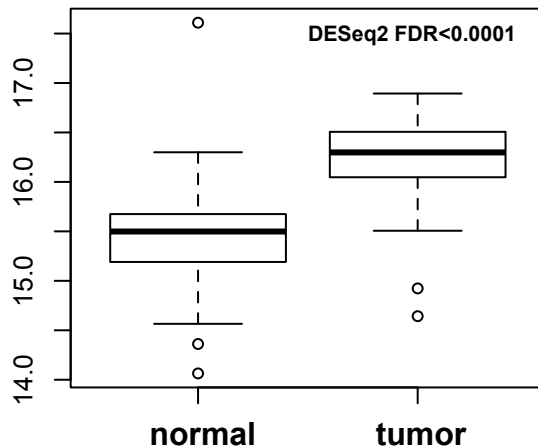

Supplement: Supplementary file 6 — Gene expression of genes in close proximity to the top diagnostic model. (PDF 118 kb) [file 12885_2017_3252_MOESM6_ESM.pdf]

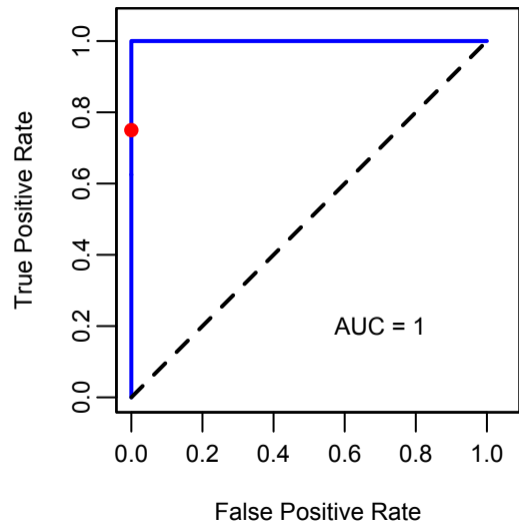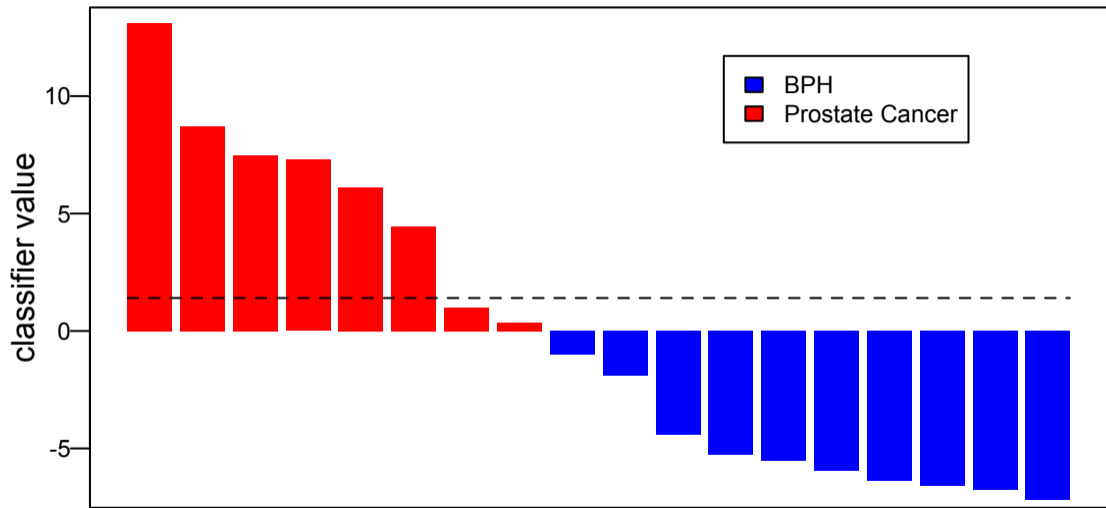

Supplement: Supplementary file 7 — ROC curve and waterfall plot for diagnostic model applied to an independent cohort of prostate cancer and benign prostate hyperplasia samples. (PDF 775 kb) [file 12885_2017_3252_MOESM7_ESM.pdf]

**cg00054525**

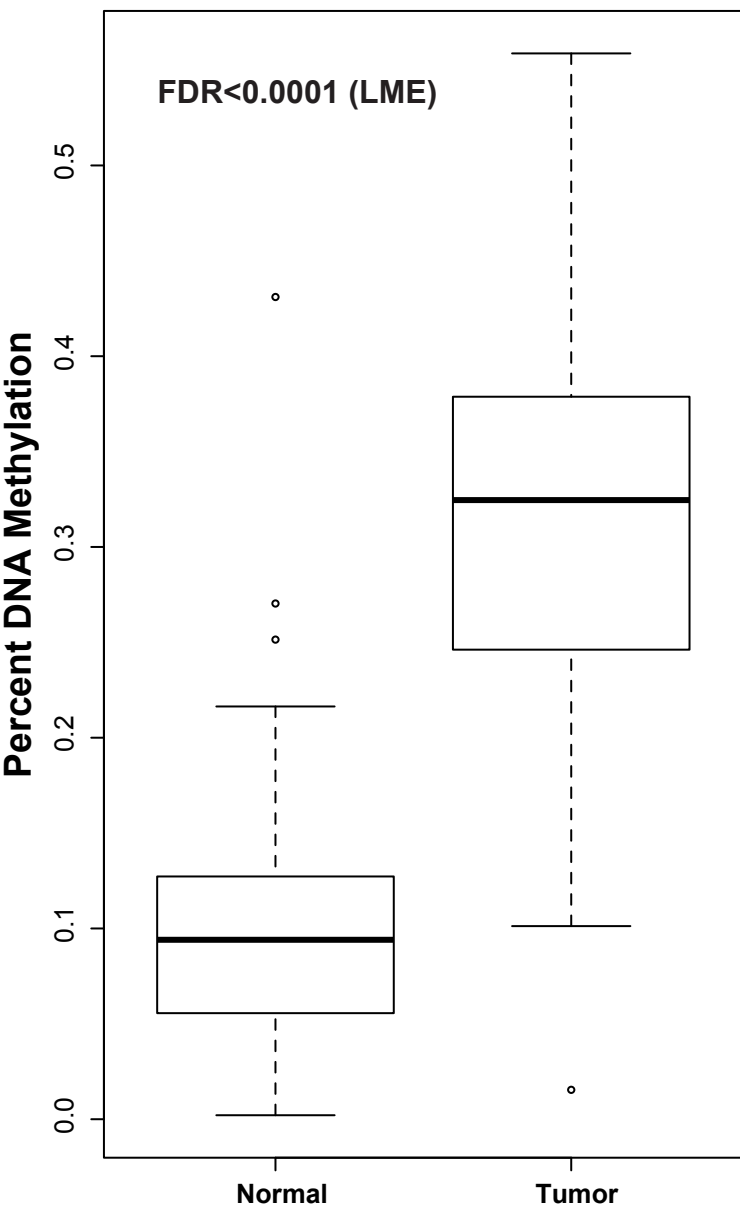

**cg14781281**

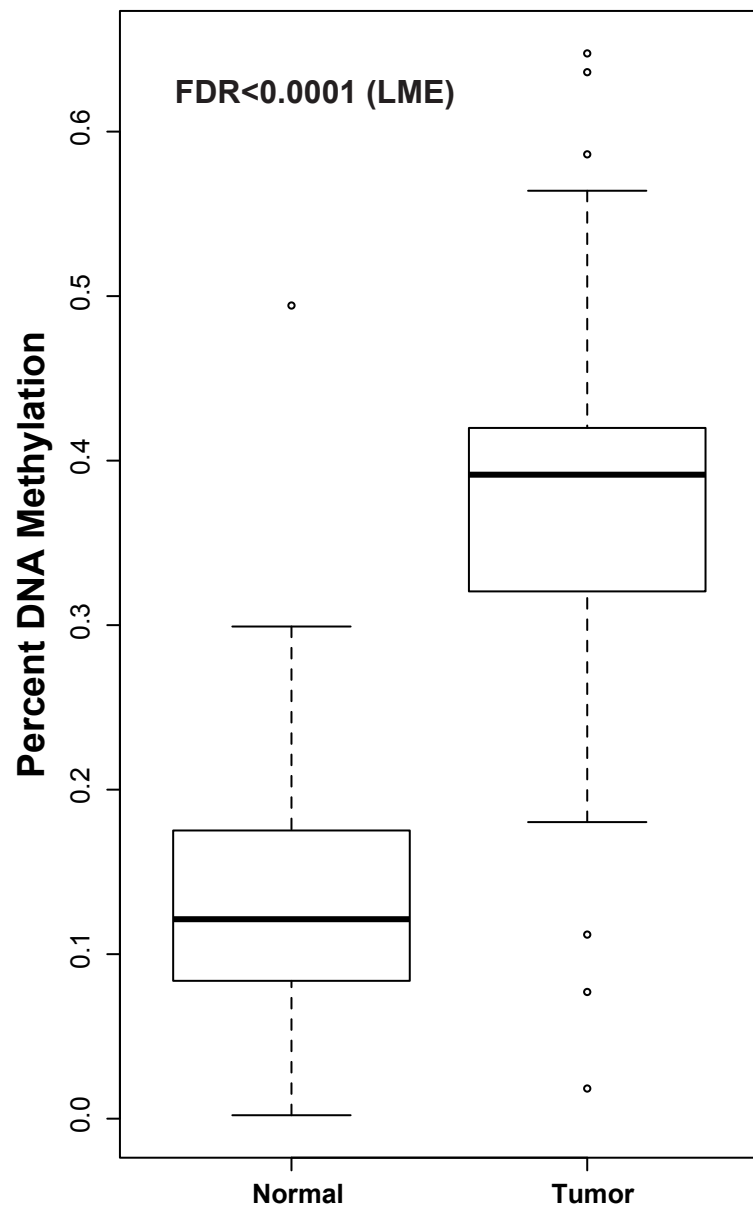

**cg15338327**

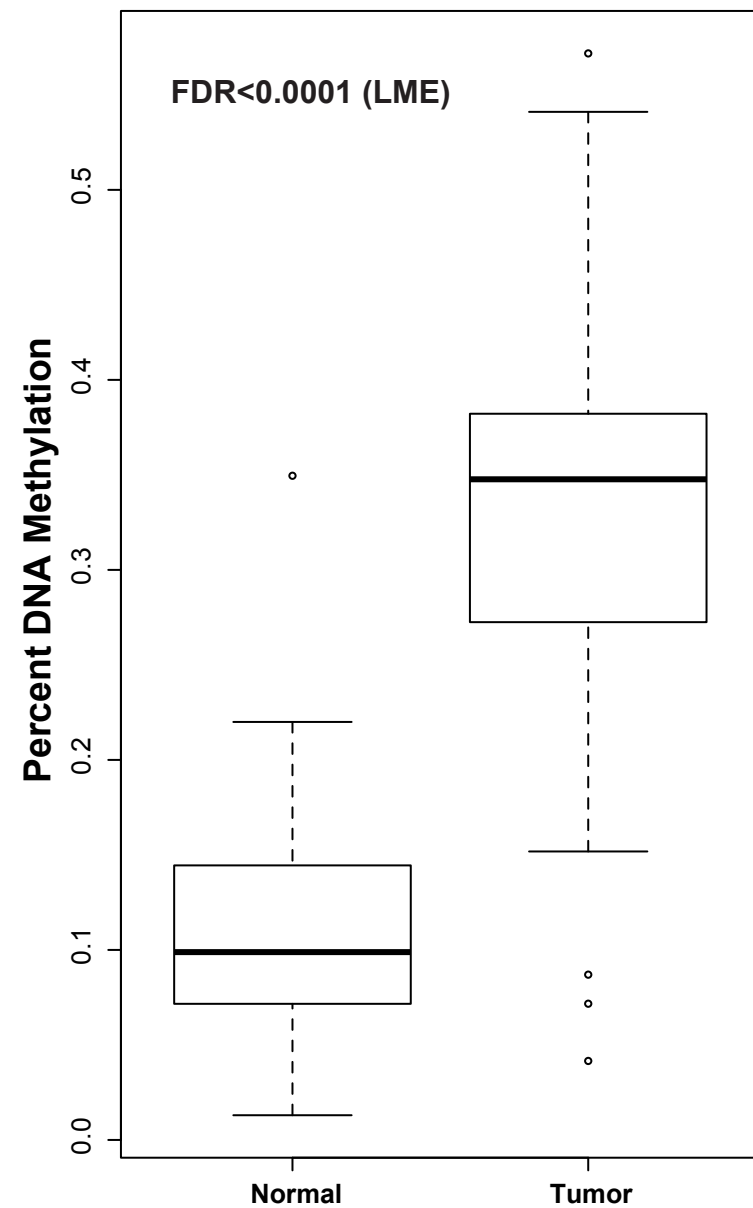

**Tissue Type**

Supplement: Supplementary file 8 — Boxplots of CpGs in the top diagnostic model from hypermethylated CpGs. Normal data is from benign-adjacent tissues and Tumor data is from patient cancer tissues. (PDF 129 kb) [file 12885_2017_3252_MOESM8_ESM.pdf]

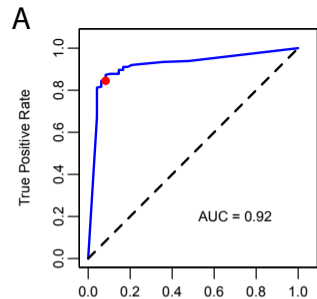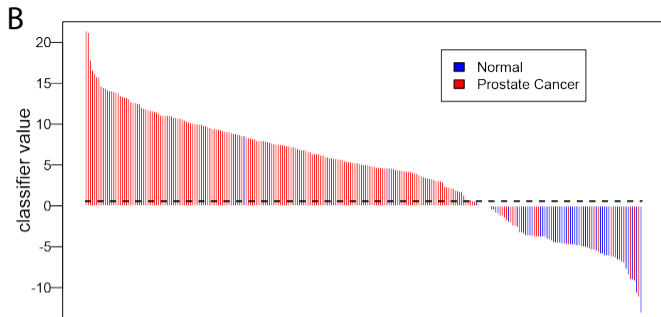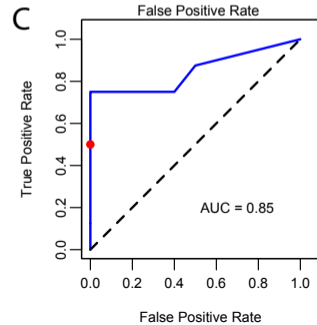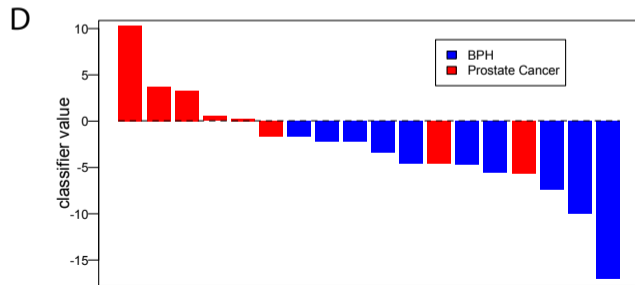

Supplement: Supplementary file 9 — ROC curve and waterfall plot for the top hypermethylated CpG model in the TCGA validation cohort (a-b) and BPH cohort (c-d). (PDF 842 kb) [file 12885_2017_3252_MOESM9_ESM.pdf]
